# Beneficial effects of muscone on cardiac remodeling in a mouse model of myocardial infarction

**DOI:** 10.3892/ijmm.2014.1766

**Published:** 2014-05-02

**Authors:** XIAOYAN WANG, HAOYU MENG, PENGSHENG CHEN, NAIQUAN YANG, XIN LU, ZE-MU WANG, WEI GAO, NINGTIAN ZHOU, MIN ZHANG, ZHIHUI XU, BO CHEN, ZHENGXIAN TAO, LIANGSHENG WANG, ZHIJIAN YANG, TIEBIN ZHU

**Affiliations:** 1 Department of Cardiology, The First Affiliated Hospital of Nanjing Medical University, Nanjing, Jiangsu 210029, P.R. China; 2 Department of Cardiology, Wuxi No. 3 Hospital Affiliated to Nantong University, Wuxi, Jiangsu 214043, P.R. China; 3 Department of Cardiology, Huai’an Second People’s Hospital Affiliated to Xuzhou Medical College, Huai’an, Jiangsu 223002, P.R. China

**Keywords:** muscone, myocardial infarction, myocardial remodeling, mouse model

## Abstract

Musk has been traditionally used in East Asia to alleviate the symptoms of angina pectoris. However, it remains unclear as to whether muscone, the main active ingredient of musk, has any beneficial effects on persistent myocardial ischemia *in vivo*. The aim of the present study was to investigate whether muscone can improve cardiac function and attenuate myocardial remodeling following myocardial infarction (MI) in mice. Mice were subjected to permanent ligation of the left anterior descending coronary artery to induce MI, and then randomly treated with muscone (2 mg/kg/day) or the vehicle (normal saline) for 3 weeks. Sham-operated mice were used as controls and were also administered the vehicle (normal saline). Treatment with muscone significantly improved cardiac function and exercise tolerance, as evidenced by the decrease in the left ventricular end-systolic diameter, left ventricular end-diastolic diameter, as well as an increase in the left ventricular ejection fraction, left ventricular fractional shortening and time to exhaustion during swimming. Pathological and morphological assessments indicated that treatment with muscone alleviated myocardial fibrosis, collagen deposition and improved the heart weight/body weight ratio. Muscone inhibited the inflammatory response by reducing the expression of transforming growth factor (TGF)-β1, tumor necrosis factor (TNF)-α, interleukin (IL)-1β and nuclear factor (NF)-κB. Treatment with muscone also reduced myocardial apoptosis by enhancing Bcl-2 and suppressing Bax expression. Muscone also induced the phosphorylation of protein kinase B (Akt) and endothelial nitric oxide synthase (eNOS). Our results demonstrate that muscone ameliorates cardiac remodeling and dysfunction induced by MI by exerting anti-fibrotic, anti-inflammatory and anti-apoptotic effects in the ischemic myocardium.

## Introduction

Myocardial infarction (MI) is one of the most important health challenges worldwide (1,2). Although the mortality rate associated with MI has decreased due to early thrombolysis, percutaneous coronary intervention or coronary artery bypass grafting, patients who survive inevitably suffer from consequent cardiac remodeling and heart failure (HF) (3–8). Thus, it is urgent to explore effective therapeutic approaches in order to prevent cardiac remodeling induced by MI.

As a highly valued traditional Chinese medicine, musk is adopted extensively by clinicians for the treatment of cardio-cerebrovascular diseases, such as angina, vascular headaches and stroke (9–17). Musk is believed to have antioxidant, anti-inflammatory, vasodilator and angiogenic effects (18,19). As the most important monomer of musk, muscone has been found in *in vitro* studies to have multiple cardioprotective effects on injury caused by myocardial ischemia/reperfusion (20). However, it remains unclear as to whether muscone is also effective in persistent myocardial ischemia *in vivo*.

It has been confirmed that myocardial fibrosis, inflammation, apoptosis and endothelial dysfunction are typical characteristics of cardiac remodeling following MI (4). Pro-inflammatory mediators, such as transforming growth factor (TGF)-β1, tumor necrosis factor (TNF)-α and interleukin (IL)-1β amplify the inflammatory response and play pathogenic roles in myocardial fibrosis and remodeling (21,22). The decreased expression of Bax protein and the increased expression of Bcl-2 inhibit apoptosis, both contributing to reserve cardiac function. The activation of the phosphatidylinositol 3-kinase (PI3K) signaling pathway, including protein kinase B (Akt) and endothelial nitric oxide synthase (eNOS) phosphorylation promote the synthesis of nitric oxide (NO), which is considered to be involved in the protection of endothelial function (16,23–25).

In light of the established pharmacological effects of muscone, we hypothesized that the administration of muscone may ameliorate cardiac remodeling and improve cardiac function following MI. In the present study, we used a mouse model of persistent myocardial ischemia by permanent ligation of the left anterior descending (LAD) coronary artery (26) in order to investigate the cardioprotective effects of muscone on MI *in vivo*, as well as the potential mechanisms involved.

## Materials and methods

### Mouse model of myocardial infarction

All animal care and experimental procedures were in accordance with the Ethics Review Committee of Nanjing Medical University (Nanjing, China) for the Use and Application of Laboratory Animals (Permit No. NJMU-ERLAUA-2012-11-01-01). C57BL/6J mice (Laboratory Animal Center of Nanjing Medical University, male, aged 6–8 weeks, weighing 18–24 g) were anesthetized with sodium pentobarbital (50 mg/kg, intraperitoneally) followed by tracheal intubation with artificial ventilation. Following anesthesia, the mice were fixed on a heating pad to maintain normothermia. A thoracotomy was then performed to expose the heart. The respiratory conditions were monitored and an electrocardiogram was performed. MI was induced by the permanent ligation of the LAD coronary artery with an 8-0 polypropylene suture passing 2–3 mm from the inferior margin of left auricle. MI was confirmed by myocardial blanching and ST segment elevation of the electrocardiogram. The sham-operated group was subjected to a similar procedure without ligating the LAD coronary artery. The thorax was closed layer by layer, and penicillin was injected intramuscularly. Following recovery of spontaneous breathing, the mice were extubated and placed on an electric blanket for recovery.

### Muscone treatment protocol

Muscone was purchased from the National Institute for the Control of Pharmaceutical and Biological Products (Beijing, China; purity, >98%; concentration, 1 g/ml). The mice that survived were randomly treated with muscone (2 mg/kg/day, intragastric administration, n=27), as previously described (17,27) or the vehicle (normal saline; intragastric administration, n=27) for 3 weeks. The sham-operated mice also received the vehicle (normal saline; n=9). The mice were observed every 12 h, weighed each week, and sacrificed by suffocation with carbon dioxide on the 21st day. The survival rate was measured by Kaplan-Meier survival curve analysis.

### Measurement of time to exhaustion during swimming

The mice were placed in a pool (temperature, 31±1°C; depth, 20 cm; surface area, 0.25 m^2^) for swimming. The time to exhaustion during swimming was defined as a consecutive sinking of >3 times. The time to exhaustion during swimming for each mouse was measured and recorded.

### Echocardiographic measurement

Cardiac structure and function on the 1st, 10st and 21st day following treatment were evaluated by using a high-frequency ultrasound system Vevo 2100 (VisualSonics, Inc., Toronto, ON, Canada) with a 30-MHz central frequency scan head. After the mice were anesthetized, two-dimensional echocardiographic measurements were obtained. The left ventricular end-systolic diameter (LVESd), left ventricular end-diastolic diameter (LVEDd), as well as the left ventricular ejection fraction (LVEF) and left ventricular fractional shortening (LVFS) were recorded.

### Measurement of myocardial fibrosis

The hearts were harvested and weighed, washed in phosphate-buffered saline, fixed in 4% paraformaldehyde overnight and embedded in paraffin. Each paraffin-embedded heart was cut into sections (4 μm thick) through the infarct area and stained with Masson’s trichrome. Each section was imaged under a microscope (Nikon, Tokyo, Japan). Fibrosis was calculated by computerized planimetry using ImageJ software, version 1.44 (NIH, Bethesda, MD, USA).

### Measurement of apoptotic cells by TUNEL staining

On the 21st day after treatment, the distribution of apoptotic cells was detected using the In Situ Cell Death Detection kit (Biunique, Nanjing, China). All the slices were stained with DAPI (1 μg/ml; Sigma, St. Louis, MO, USA) for the assessment of nuclear morphology. The FITC-labeled TUNEL-positive cells were imaged under a fluorescence microscope at a magnification of ×400 (Nikon) and 3 horizons were randomly selected in the marginal zone of infarction from each slice. The FITC-labeled TUNEL-positive cells were counted using Image-Pro Plus software (Media Cybernetics, Rockville, MD, USA) and the apoptotic index was calculated as follows: apoptotic cell number/1,000 cells ×100%.

### Western blot analysis

Total protein was obtained from left ventricular myocardial tissues by sonication, centrifugation and heat denaturation. The protein lysates were electrophoresed and separated on 6–12% SDS-PAGE and transferred onto nitrocellulose membranes (Bio-Rad, Hercules, CA, USA). The membranes were blocked with 5% skim milk at room temperature for 1 h, and then incubated overnight at 4°C with primary antibodies, including rabbit anti-Akt (1:1,000; Cell Signaling Technology, Inc., Danvers, MA, USA), rabbit anti-phospho Akt (1:1,000; Cell Signaling Technology, Inc.), rabbit anti-eNOS (1:1,000; Sigma), rabbit anti-phospho-eNOS (1:200; Santa Cruz Biotechnology, Inc., Santa Cruz, CA, USA), rabbit anti-NF-κB (1:800; Cell Signaling Technology, Inc.), rabbit anti-Bcl-2 (1:800; BioWorld, Inc., Visalia, CA, USA), rabbit anti-Bax (1:800; BioWorld, Inc.), and rabbit anti-GAPDH (1:1,000; Cell Signaling Technology, Inc.). The membranes were then incubated with HRP-conjugated secondary antibodies (1:500; Santa Cruz Biotechnology, Inc.) at room temperature for 1 h. The SuperSignal ECL kit (Thermo Fisher Scientific, Rockville, MD, USA) was used to detect the antigen-antibody complexes in a western blotting detection system (Bio-Rad). The results were expressed as density values normalized to GAPDH.

### ELISA measurement of TGF-β1, TNF-α and IL-1β

The levels of TGF-β1, TNF-α and IL-1β (Bio-Swamp, Shanghai, China) in the left ventricular myocardium were measured by ELISA. Myocardial samples (20 mg) were homogenized in 200 μl of 1× phosphate-buffered saline (pH 7.4), then stored overnight at −20°C. The homogenates were determined by 2 freeze-thaw cycles to break the cell membranes followed by centrifugation at 5000 × g for 10 min. The samples were then tested immediately following the procedure recommended by the manufacturer.

### Statistical analysis

The GraphPad Prism 5 Demo and SPSS 13.0 software were used for statistical analyses and graphing. The Student’s t-test or one-way ANOVA were used to evaluate the mean difference (MD) ± standard error of the mean (SEM) and 95% confidence interval (CI) of the values of the parameters between the groups. Kaplan-Meier survival analysis was performed to evaluate the overall survival of the mice following the induction of MI by the log-rank test, and a two-sided value of P<0.05 was considered to indicate a statistically significant difference.

## Results

### Treatment with muscone improves the survival rate and time to exhaustion during swimming

**Figure 1:**
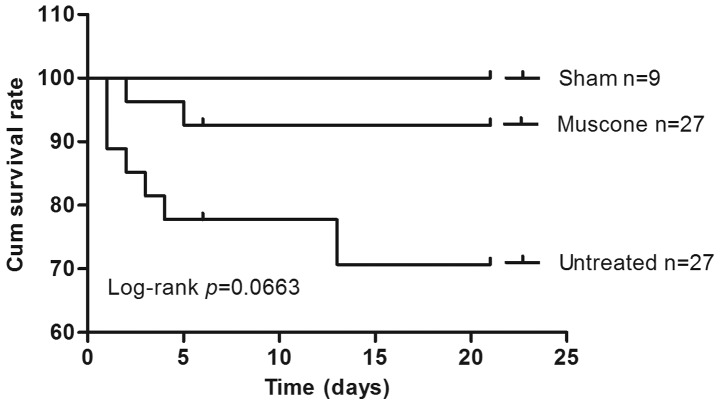
Survival rate at 3 weeks follwoing myocardial infarction (MI). Kaplan-Meier analysis revealed a trend of lower mortality in the muscone-treated mice compared with the untreated mice (log-rank, P=0.066). Sham, sham-operated group.

Compared to the untreated mice, the muscone-treated mice showed an improved survival rate [93 vs. 74%; hazard ration (HR) 0.29; 95% CI 0.07745–1.087; P=0.066] (Fig. 1). A post-mortem examination indicated that the main cause of death was HF. The time to exhaustion during swimming was reduced in the mice with MI compared to the sham-operated mice (18.70±0.5867 min vs. 28.21±0.4911 min; MD −9.515±0.9072; P<0.0001), which was improved following treatment with muscone compared with the untreated mice with MI (20.71±0.7342 min vs. 18.70±0.5867 min; MD 2.014±0.9756; 95% CI 0.004332–4.024; P=0.0495).

### Treatment with muscone improves cardiac structure and function

**Figure 2:**
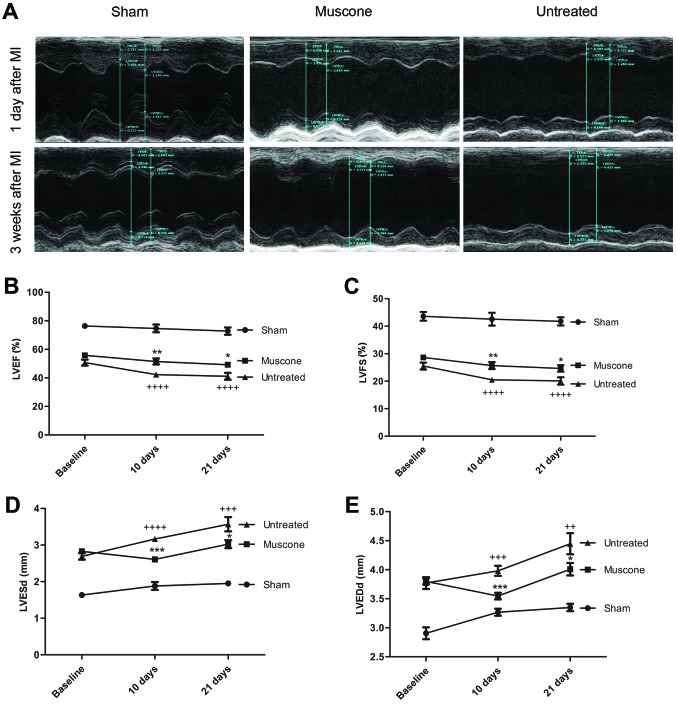
Effects of muscone on cardiac structure and function in mice with myocardial infarction (MI). (A) Representative echocardiography images of mice. Analysis of (B) left ventricular ejection fraction (LVEF), (C) left ventricular fractional shortening (LVFS), (D) left ventricular end-systolic diameter (LVESd)and (E) left ventricular end-diastolic diameter (LVEDd) at 1, 10 and 21 days following the induction of MI. ^*^P<0.05 vs. untreated group, ^**^P<0.01 vs. untreated group, ^***^P<0.001 vs. untreated group, ^++^P<0.01 vs. sham-operated group (sham), ^+++^P<0.001 vs. sham-operated group, ^++++^P<0.0001 vs. sham-operated group.

No significant difference in cardiac function was observed between the muscone-treated and untreated group at baseline. On the 21st day following the induction of MI, LVESd and LVEDd were higher, while LVFS and LVEF were lower in the mice with MI compared to the sham-operated group (P<0.05). Following treatment for 3 weeks, LVESd (3.025±0.1089 vs. 3.570±0.1965 mm; MD −0.5451±0.2246; 95% CI −1.011 to −0.07918; P=0.0239) and LVEDd (4.008±0.1073 vs. 4.447±0.1822 mm; MD −0.4398±0.2114; 95% CI −0.8782 to −0.001284; P=0.0494) were significantly lower compared to the untreated group, while LVEF (49.23±1.906 vs. 41.12±2.441%; MD 8.112±3.097; 95% CI 1.688–14.54; P=0.0157) and LVFS (24.65±1.159 vs. 20.10±1.347%; MD 4.551±1.777; 95% CI 0.8654–8.237; P=0.0178) were significantly higher in the muscone-treated group compared with the untreated group (Fig. 2A–E). In addition, the heart weight/body weight ratio was also higher in the mice with MI compared to the sham-operated group (P<0.01), which was reduced following treatment with muscone compared with the untreated mice (0.6120±0.01866 vs. 0.6939±0.02428; MD −0.08195±0.03062; 95% CI −0.1455 to −0.01844; P=0.0138).

### Treatment with muscone reduces myocardial fibrosis

**Figure 3:**
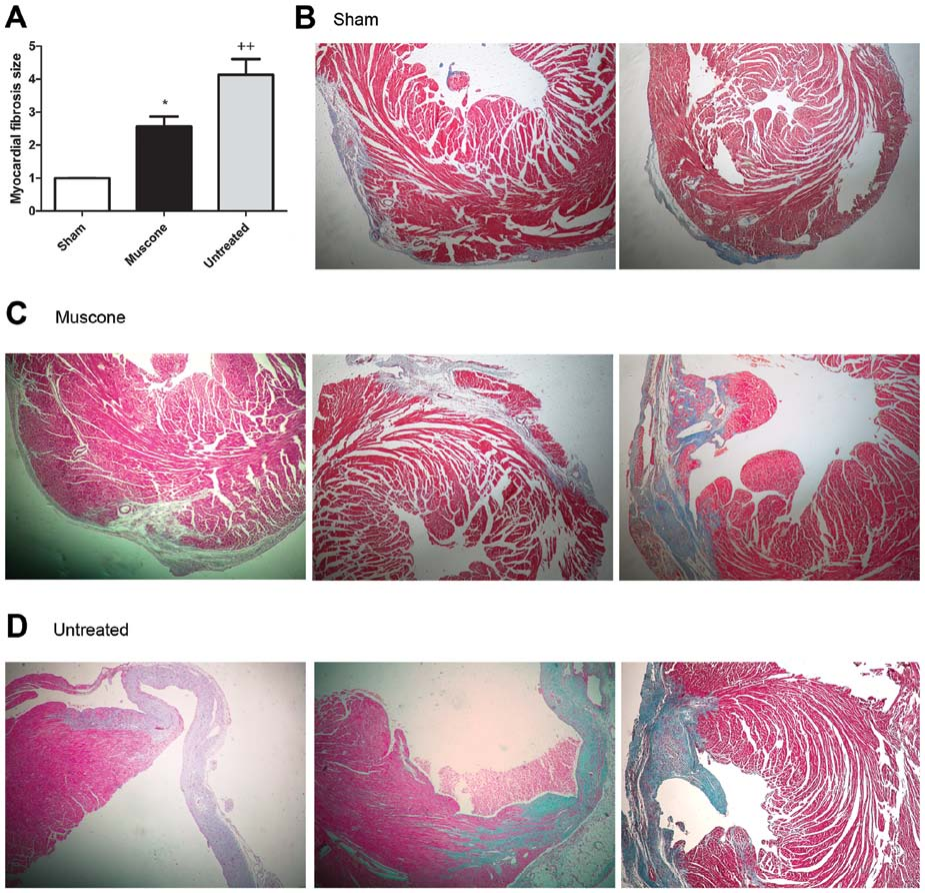
Treatment with muscone prevents myocardial fibrosis in mcie with myocardial infarction (MI). (A) Analysis of myocardial fibrosis in mice in the sham-operated group (sham), muscone-treated group and untreated group. Representative images of Masson’s trichrome-stained hearts from the (B) sham-operated mice, (C) muscone-treated mice and (D) untreated mice. Blue color represents the region with fibrosis. ^*^P<0.05 vs. untreated group, ^++^P<0.01 vs. sham-operated group.

Masson’s trichrome staining was used to detect myocardial fibrosis. The results revealed that the fibrotic area was significantly increased in the mice with MI compared to the sham-operated group (P<0.05); treatment with muscone significantly decreased myocardial fibrosis and collagen deposition compared to the untreated group (2.567±0.3032 vs. 4.136±0.4741; MD −1.568±0.5628; 95% CI −2.822 to −0.3144; P=0.0192) (Fig. 3).

### Treatment with muscone suppresses the expression of inflammatory cytokines

**Figure 4:**
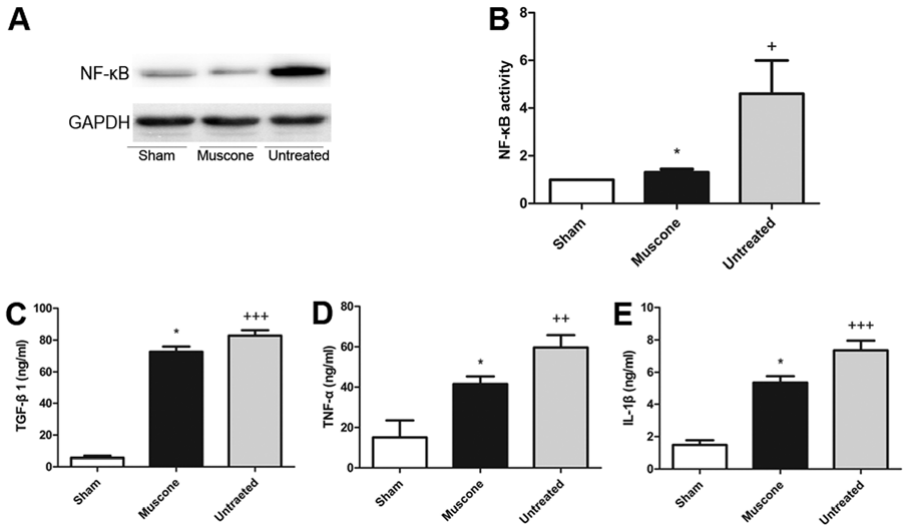
Treatment with muscone decreases the expression of inflammation cytokines following myocardial infarction (MI). (A) Western blot analysis of NF-κB. GAPDH was used an internal control. (B) Densitometric analysis of NF-κB expression normalized to GAPDH. (C) Quantitative analysis of TGF-β1, (D) TNF-α and (E) IL-1β expression in the left ventricular myocardium by ELISA, n=8 per group. ^*^P<0.05 vs. untreated group, ^+^P<0.05 vs. sham-operated group (sham), ^++^P<0.01 vs. sham-operated group, ^+++^P<0.001 vs. sham-operated group.

Compared to the sham-operated mice, the expression levels of TGF-β1, TNF-α, IL-1β and NF-κB were higher in the untreated group (P<0.05), indicating that MI upregulated the levels of pro-inflammatory cytokines. The muscone-treated group showed significantly lower expression levels of TGF-β1 (72.56±3.302 vs. 109.0±12.74; MD −36.41±12.25; 95% CI −63.36 to −9.452; P=0.0127), TNF-α (41.56±3.802 vs. 59.66±6.164l MD −18.09±7.011; 95% CI −33.53 to −2.665; P=0.0255), IL-1β (5.354±0.3977 vs. 7.345±0.6096; MD −1.990±0.7075; 95% CI −3.548 to −0.4330; P=0.0169) and NF-κB (1.321±0.1247 vs. 4.817±1.185; MD −3.496±1.192; 95% CI −6.804 to −0.1870; P=0.0427) compared to the untreated group (Fig. 4).

### Treatment with muscone decreases myocardial apoptosis

**Figure 5:**
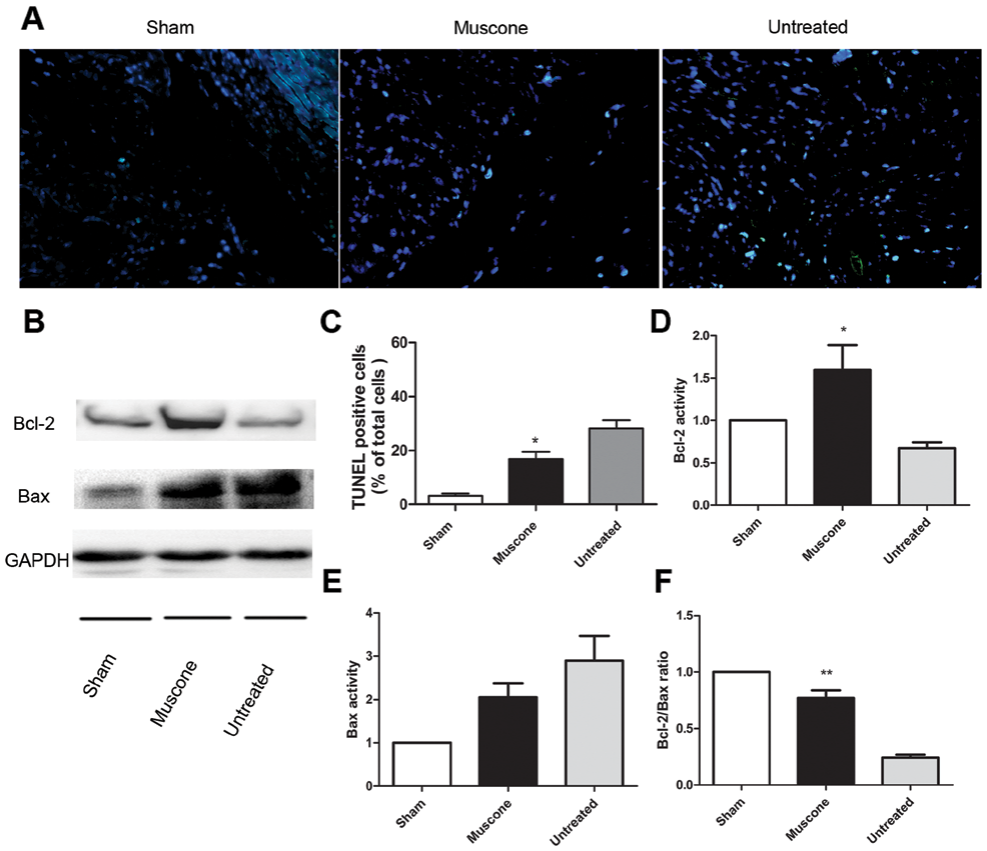
Anti-apoptotic effects of muscone on myocardial cells. (A) TUNEL analysis of apoptotic myocardial cells from sham-operated mice, muscone treated mice and untreated mice at 3 weeks following the induciton of myocardial infarction (MI). TUNEL-positive cells appear green, DAPI-counterstained nuclei are blue. (B) Western blot analysis of Bcl-2 and Bax. GAPDH was used as an internal control. (C) Cardiomyocyte apoptotic index was significantly lower in the muscone-treated group compared with the untreated group. (D) Densitometric analysis of Bcl-2 expression normalized to GAPDH. (E) Densitometric analysis of Bax expression normalized to GAPDH. (F) Analysis of the ratio between the expression of Bcl-2 and Bax, n=3 per group. ^*^P<0.05 vs. untreated group, ^**^P<0.01 vs. untreated group. Sham, sham-operated group.

Compared to the sham-operated group, the untreated group showed a significantly higher apoptotic index in the marginal zone of the nfarcted myocardium (P<0.05). The apoptotic index was significantly lower in the muscone treated group compared to the untreated group (16.80±2.672 vs. 28.20±2.973; MD −11.40±3.997; 95% CI −20.62 to −2.182; P=0.0214) (Fig. 5A and B). The expression level of Bcl-2 (1.594±0.2966 vs. 0.6756±0.06635; MD 0.9180±0.3039; 95% CI 0.07424–1.762; P=0.0392) and the Bcl-2/Bax ratio (0.7715±0.06642 vs. 0.2418±0.02536; MD 0.5297±0.07110; 95% CI 0.3323–0.7271; P=0.0017) were significantly increased in the treated group compared to the untreated group (Fig. 5C–F).

### Treatment with muscone activates Akt-eNOS signaling

**Figure 6:**
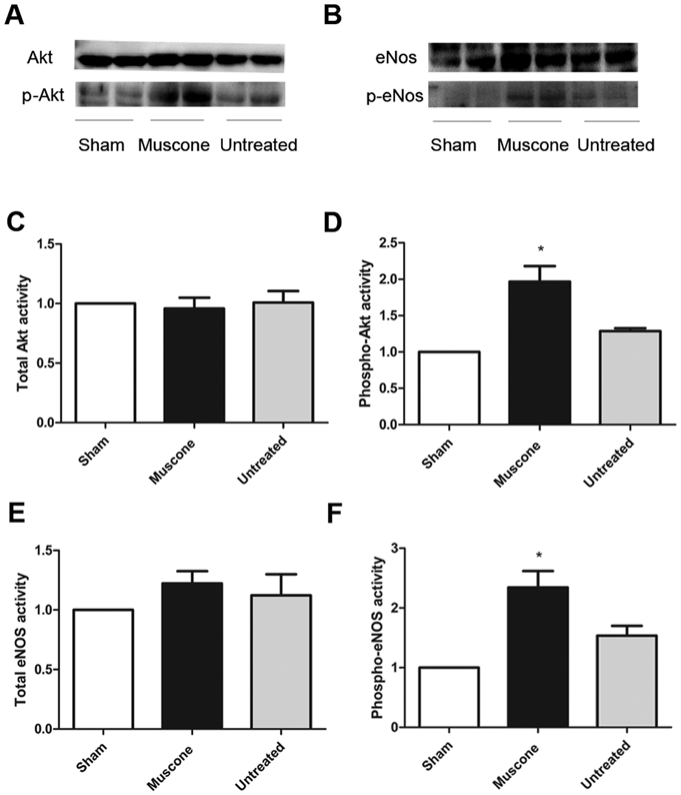
Phospho-Akt and phospho-eNOS protein expression in the left ventricular myocardium of mice. (A) Western blot analysis of total Akt, phosphor-Akt (p-Akt) and (B) total eNOS and phosphor-eNOS (p-eNOS) expression. Densitometric analysis of (C) total Akt, (D) phospho-Akt, (E) total eNOS and (F) phospho-eNOS expression, n=6 per group; ^*^P<0.05 vs. untreated group

The expression of total Akt and eNOS in the ischemic myocardium was similar among the 3 groups. However, the expression levels of phosphorylated Akt and phosphorylated eNOS were significantly lower in the mice with MI compared with the sham-operated group (P<0.05); treatment with muscone increased the phosphorylation of Akt (1.968±0.2110 vs. 1.286±0.04034; MD 0.6825±0.2149; 95% CI 0.08610–1.279; P=0.0336) and phosphorylated eNOS (2.347±0.2729 vs. 1.537±0.1645; MD 0.8095±0.3186; 95% CI 0.02995–1.589; P=0.044) compared to the untreated group (Fig. 6).

## Discussion

In this study, we investigated the cardioprotective effects of muscone in mice following the induction of MI. The key findings were as follows: i) muscone improved the survival rate and exercise tolerance, as well as the cardiac structure and function; ii) muscone attenuated myocardial fibrosis and inflammation following MIl iii) muscone exerted anti-apoptotic effects by increasing Bcl-2 and decreasing Bax expression; iv) muscone increased the phosphorylation of Akt-eNOS. Our results demonstrated that muscone is effective in preventing the progression of cardiac remodeling following MI.

Inflammatory cytokines, including TGF-β1, TNF-α and IL-1β, play crucial roles in myocardial fibrosis and the pathological progression of left ventricular remodeling following MI (28–30). NF-κB is a key transcription factor, which induces the expression of a number of target genes, including pro-inflammatory cytokines, endothelial adhesion molecules and oxidative-stress related enzymes (31). Activated NF-κB increases the expression of TGF-β1, TNF-α and IL-1β, which subsequently activates collagen deposition and myocardial fibrosis that lead to myocardial remodeling and HF (32,33). Morishita *et al* (34) reported that the inhibition of NF-κB binding decreased cardiac damage following MI. In the present study, we also observed the increased expression of TGF-β1, TNF-α, IL-1β and NF-κB following the induction of MI, and treatment with muscone significantly decreased the expression of these inflammatory markers, which indicated that the cardioprotective effects of muscone may be attributed to its anti-inflammation effects.

Apoptosis plays an important role in MI and HF. Previous studies have demonstrated that ischemia-induced apoptosis and necrosis contribute to autophagic cardiomyocyte death and cardiomyocyte loss in myocardial ischemic injury (35,36). Apoptosis is characterized by the increased expression of pro-apoptotic Bax family proteins and the decreased expression of anti-apoptotic Bcl-2 family proteins (7). Grimm *et al* (38) proved that Bcl-2 also inhibited the activity of NF-κB. It has also been demonstrated that the amplification of apoptosis is sufficient to increase fibrosis, indicating a critical role for cardiomyocyte apoptosis in the progression of myocardial fibrosis (39). Inflammatory cytokines, such as TNF-α and IL-1β also induce apoptosis and contribute to the process of myocardial remodeling (21,36). Consistently, we demonstrated that the apoptotic level in the marginal zone of the infarcted myocardium was decreased by treatment with muscone. Furthermore, our results also revealed the upregulated expression of Bcl-2 and the downregulated expression of Bax in the muscone-treated group. Thus, we hypothesized that treatment with muscone reduced cardiac remodeling due to its anti-apoptotic effects.

The balance of eNOS activity is the hallmark of vascular endothelial function, such as endothelium-dependent relaxation, the integrity of the vascular endothelium and angiogenesis (40,41). The activation of eNOS promotes the synthesis of NO, which then protects the ischemic heart by regulating vascular remodeling and angiogenesis (42,43). It has been shown that the activation of the PI3K-Akt-eNOS pathway alleviates cardiac ischemia-reperfusion injury (44,45), while eNOS deficiency causes myocardial apoptosis and HF (46). In our study, we observed that the administration of muscone significantly induced the phosphorylation of Akt and eNOS, indicating that treatment with muscone may exert protective effects on the ischemic myocardium by activating the PI3K-Akt-eNOS pathway.

There were some limitations of the present study. Firstly, although treatment with muscone led to a marked improvement in left ventricular morphology and function, our study investigated only some of its possible mechanisms of action. Further studies are required to reveal additional pathways. Secondly, the observation period was relatively short. Thirdly, whether muscone can function in conjunction with other medications to achieve better therapeutic effects needs to be further explored.

In conclusion, in the present study, we identified that the administration of muscone has notable benefits, preventing cardiac remodeling following MI. The potential mechanisms may be associated with the anti-fibrotic, anti-inflammatory and anti-apoptotic effects of muscone on the ischemic myocardium. Given our increased understanding of muscone and cardiovascular diseases, muscone is highly valued as a novel therapeutic application in myocardial ischemic injury following MI.

## References

[1] Lin DL, Chang HC, Huang SH (2004). Characterization of allegedly musk-containing medicinal products in Taiwan. J Forensic Sci.

[2] Go AS, Mozaffarian D, Roger VL, Benjamin EJ, Berry JD, Borden WB, Bravata DM, Dai S, Ford ES, Fox CS, Franco S, Fullerton HJ, Gillespie C, Hailpern SM, Heit JA, Howard VJ, Huffman MD, Kissela BM, Kittner SJ, Lackland DT, Lichtman JH, Lisabeth LD, Magid D, Marcus GM, Marelli A, Matchar DB, McGuire DK, Mohler ER, Moy CS, Mussolino ME, Nichol G, Paynter NP, Schreiner PJ, Sorlie PD, Stein J, Turan TN, Virani SS, Wong ND, Woo D, Turner MB (2013). Heart disease and stroke statistics--2013 update: a report from the American Heart Association. Circulation.

[3] Hung J, Teng TH, Finn J, Knuiman M, Briffa T, Stewart S, Sanfilippo FM, Ridout S, Hobbs M (2013). Trends from 1996 to 2007 in incidence and mortality outcomes of heart failure after acute myocardial infarction: a population-based study of 20,812 patients with first acute myocardial infarction in Western Australia. J Am Heart Assoc.

[4] Sun Y, Zhang JQ, Zhang J, Lamparter S (2000). Cardiac remodeling by fibrous tissue after infarction in rats. J Lab Clin Med.

[5] See F, Kompa A, Martin J, Lewis DA, Krum H (2005). Fibrosis as a therapeutic target post-myocardial infarction. Curr Pharm Des.

[6] Tucci PJ (2011). Pathophysiological characteristics of the post-myocardial infarction heart failure model in rats. Arq Bras Cardiol.

[7] Rohde LE, Ducharme A, Arroyo LH, Aikawa M, Sukhova GH, Lopez-Anaya A, McClure KF, Mitchell PG, Libby P, Lee RT (1999). Matrix metalloproteinase inhibition attenuates early left ventricular enlargement after experimental myocardial infarction in mice. Circulation.

[8] Lindsey ML, Mann DL, Entman ML, Spinale FG (2003). Extracellular matrix remodeling following myocardial injury. Ann Med.

[9] Yan SK, Zhang WD, Liu RH, Zhan YC (2006). Chemical fingerprinting of Shexiang Baoxin Pill and simultaneous determination of its major constituents by HPLC with evaporative light scattering detection and electrospray mass spectrometric detection. Chem Pharm Bull (Tokyo).

[10] Shen W, Fan WH, Shi HM (2010). Effects of shexiang baoxin pill on angiogenesis in atherosclerosis plaque and ischemic myocardium. Zhongguo Zhong Xi Yi Jie He Za Zhi.

[11] Fan X, Shi M, Wang Y, Liang Q, Luo G (2011). Transcriptional profiling analysis of HMP-treated rats with experimentally induced myocardial infarction. J Ethnopharmacol.

[12] Wu DJ, Hong HS, Jiang Q (2005). Effect of shexiang baoxin pill in alleviating myocardial fibrosis in spontaneous hypertensive rats. Zhongguo Zhong Xi Yi Jie He Za Zhi.

[13] Cai YM, He Y, Qiu T, Zou J, Sun DP, Peng QH, Jia RX, Zhao HR (2011). Research on frequency of application with modern Chinese herbal medicine. Chin J Integr Med.

[14] Wang LJ, Luo XP, Wang Y (2008). Evaluation on tolerability and safety of long-term administration with shexiang baoxin pill in patients with coronary heart disease of stable angina pectoris. Zhongguo Zhong Xi Yi Jie He Za Zhi.

[15] Wang S, Zheng Z, Weng Y, Yu Y, Zhang D, Fan W, Dai R, Hu Z (2004). Angiogenesis and anti-angiogenesis activity of Chinese medicinal herbal extracts. Life Sci.

[16] Xiang L, Jiang P, Zhan C, Chen Z, Liu X, Huang X, Wang S, Hu Y, Zhang W, Liu R (2012). The serum metabolomic study of intervention effects of the traditional Chinese medicine Shexiang Baoxin Pill and a multi-component medicine polypill in the treatment of myocardial infarction in rats. Mol Biosyst.

[17] Sun R, Zhang ZP, Huang W, Lv LP, Ren HY (2009). Protective effects of muskone on rats with complete cerebral ischemia. Trad Chin Drug Res Clin Pharmacol.

[18] Wei G, Chen DF, Lai XP, Liu DH, Deng RD, Zhou JH, Zhang SX, Li YW, Li H, Zhang QD (2012). Muscone exerts neuroprotection in an experimental model of stroke via inhibition of the fas pathway. Nat Prod Commun.

[19] Tanaka E, Funae Y, Imaoka S, Misawa S (1991). Characterization of liver microsomal cytochrome P450 from rats treated with muscone (3-methylcyclopentadecanone). Biochem Pharmacol.

[20] Wu Q, Li H, Wu Y, Shen W, Zeng L, Cheng H, He L (2011). Protective effects of muscone on ischemia-reperfusion injury in cardiac myocytes. J Ethnopharmacol.

[21] Hori M, Nishida K (2009). Oxidative stress and left ventricular remodelling after myocardial infarction. Cardiovasc Res.

[22] Vilahur G, Juan-Babot O, Pena E, Onate B, Casani L, Badimon L (2011). Molecular and cellular mechanisms involved in cardiac remodeling after acute myocardial infarction. J Mol Cell Cardiol.

[23] Yang C, Talukder MA, Varadharaj S, Velayutham M, Zweier JL (2013). Early ischaemic preconditioning requires Akt- and PKA-mediated activation of eNOS via serine1176 phosphorylation. Cardiovasc Res.

[24] Chen LL, Zhu TB, Yin H, Huang J, Wang LS, Cao KJ, Yang ZJ (2010). Inhibition of MAPK signaling by eNOS gene transfer improves ventricular remodeling after myocardial infarction through reduction of inflammation. Mol Biol Rep.

[25] Wu JX, Liang C, Ren YS (2009). Effects of shexiang baoxin pill on function and nitric oxide secretion of endothelial progenitor cells. Zhongguo Zhong Xi Yi Jie He Za Zhi.

[26] Salto-Tellez M, Yung Lim S, El-Oakley RM, Tang TP, Za AL, Lim SK (2004). Myocardial infarction in the C57BL/6J mouse: a quantifiable and highly reproducible experimental model. Cardiovasc Pathol.

[27] Liang H, Luo BY (2005). The study of muscone on attenuating excitotoxicity during acute cerebral ischemia. Zhong Yao Yao Li Yu Lin Chuang.

[28] Vivar R, Humeres C, Ayala P, Olmedo I, Catalan M, Garcia L, Lavandero S, Diaz-Araya G (1832). TGF-beta1 prevents simulated ischemia/reperfusion-induced cardiac fibroblast apoptosis by activation of both canonical and non-canonical signaling pathways. Biochim Biophys Acta.

[29] Gu Q, Yang XP, Bonde P, DiPaula A, Fox-Talbot K, Becker LC (2006). Inhibition of TNF-alpha reduces myocardial injury and proinflammatory pathways following ischemia-reperfusion in the dog. J Cardiovasc Pharmacol.

[30] Siwik DA, Chang DL, Colucci WS (2000). Interleukin-1beta and tumor necrosis factor-alpha decrease collagen synthesis and increase matrix metalloproteinase activity in cardiac fibroblasts in vitro. Circ Res.

[31] Stephenson D, Yin T, Smalstig EB, Hsu MA, Panetta J, Little S, Clemens J (2000). Transcription factor nuclear factor-kappa B is activated in neurons after focal cerebral ischemia. J Cereb Blood Flow Metab.

[32] Ogawa K, Chen F, Kuang C, Chen Y (2004). Suppression of matrix metalloproteinase-9 transcription by transforming growth factor-beta is mediated by a nuclear factor-kappaB site. Biochem J.

[33] Hirotani S, Otsu K, Nishida K, Higuchi Y, Morita T, Nakayama H, Yamaguchi O, Mano T, Matsumura Y, Ueno H, Tada M, Hori M (2002). Involvement of nuclear factor-kappaB and apoptosis signal-regulating kinase 1 in G-protein-coupled receptor agonist-induced cardiomyocyte hypertrophy. Circulation.

[34] Morishita R, Sugimoto T, Aoki M, Kida I, Tomita N, Moriguchi A, Maeda K, Sawa Y, Kaneda Y, Higaki J, Ogihara T (1997). In vivo transfection of cis element ‘decoy’ against nuclear factor-kappaB binding site prevents myocardial infarction. Nat Med.

[35] Elsasser A, Vogt AM, Nef H, Kostin S, Mollmann H, Skwara W, Bode C, Hamm C, Schaper J (2004). Human hibernating myocardium is jeopardized by apoptotic and autophagic cell death. J Am Coll Cardiol.

[36] Crow MT, Mani K, Nam YJ, Kitsis RN (2004). The mitochondrial death pathway and cardiac myocyte apoptosis. Circ Res.

[37] Kluck RM, Bossy-Wetzel E, Green DR, Newmeyer DD (1997). The release of cytochrome c from mitochondria: a primary site for Bcl-2 regulation of apoptosis. Science.

[38] Grimm S, Bauer MK, Baeuerle PA, Schulze-Osthoff K (1996). Bcl-2 down-regulates the activity of transcription factor NF-kappaB induced upon apoptosis. J Cell Biol.

[39] Syed FM, Hahn HS, Odley A, Guo Y, Vallejo JG, Lynch RA, Mann DL, Bolli R, Dorn GW (2005). Proapoptotic effects of caspase-1/interleukin-converting enzyme dominate in myocardial ischemia. Circ Res.

[40] Sharma S, Singh M, Sharma PL (2013). Mechanism of hyperhomocysteinemia-induced vascular endothelium dysfunction - possible dysregulation of phosphatidylinositol-3-kinase and its downstream phosphoinositide dependent kinase and protein kinase B. Eur J Pharmacol.

[41] Yasuda S, Kobayashi H, Iwasa M, Kawamura I, Sumi S, Narentuoya B, Yamaki T, Ushikoshi H, Nishigaki K, Nagashima K, Takemura G, Fujiwara T, Fujiwara H, Minatoguchi S (2009). Antidiabetic drug pioglitazone protects the heart via activation of PPAR-gamma receptors, PI3-kinase, Akt, and eNOS pathway in a rabbit model of myocardial infarction. Am J Physiol Heart Circ Physiol.

[42] Fulton D, Gratton JP, McCabe TJ, Fontana J, Fujio Y, Walsh K, Franke TF, Papapetropoulos A, Sessa WC (1999). Regulation of endothelium-derived nitric oxide production by the protein kinase Akt. Nature.

[43] Bell RM, Yellon DM (2003). Bradykinin limits infarction when administered as an adjunct to reperfusion in mouse heart: the role of PI3K, Akt and eNOS. J Mol Cell Cardiol.

[44] Tsutsumi YM, Tsutsumi R, Mawatari K, Nakaya Y, Kinoshita M, Tanaka K, Oshita S (2011). Compound K, a metabolite of ginsenosides, induces cardiac protection mediated nitric oxide via Akt/PI3K pathway. Life Sci.

[45] Balakumar P, Kathuria S, Taneja G, Kalra S, Mahadevan N (2012). Is targeting eNOS a key mechanistic insight of cardiovascular defensive potentials of statins?. J Mol Cell Cardiol.

[46] Zhao X, Lu X, Feng Q (2002). Deficiency in endothelial nitric oxide synthase impairs myocardial angiogenesis. American journal of physiology Am J Physiol Heart Circ Physiol.

